# The role of serum biomarkers in the diagnosis and prognosis of oral cancer: A systematic review

**DOI:** 10.4317/jced.52736

**Published:** 2016-04-01

**Authors:** Ana Fernández-Olavarría, Regina Mosquera-Pérez, Rosa-María Díaz-Sánchez, Maria-Angeles Serrera-Figallo, José-Luis Gutiérrez-Pérez, Daniel Torres-Lagares

**Affiliations:** 1 DDS.School of Dentistry. University of Seville; 2 PhD, DDS. School of Dentistry. University of Seville; 3 DMD. Professor of Oral Surgery. Chairman of Oral Surgery. Department of Stomatology. University of Seville; 4 PhD, DDS, MSc (Oral Surgery). Proffesor of Oral Surgery. Department of Stomatology. University of Seville

## Abstract

**Introduction:**

Oral cancer is one of the causes of major morbidity and mortality in the world although incidence varies in the different geographical locations and races. Advances in molecular biology and cancer research have allowed elucidating serum biomarkers to improve diagnostic methods. The aim of this article systematic review is to highlight the utility and clinical value of serum biomarkers in the diagnosis and prognosis of oral cancer.

**Material and Methods:**

A systematic literature review using PubMed (MEDLINE databases) revealed a total of 140 articles related to this topic. Of those articles, 29 were included in the final review. We included articles published in English in the last five years, developed in human as cases and controls studies, retrospective or prospective studies and specific studies that analyzed a certain biomarker in serum.

**Results:**

All of the studies include in this systematic review found significant differences in patients. Of those articles included, 2 used biomarkers to determinate cancerous phenotype, 11 mentioned their results were associated with worse prognosis and overall survival, 4 correlated biomarker concentration to clinical stages, 4 concluded it could be a helpful in diagnosis and 8 studies did not find a clear utility of the analysed biomarker. Due to differences in the presentation of data, meta-analysis was not possible.

**Conclusions:**

Biomarker use for diagnosis and prognosis is supported by clinical and scientific evidence is relevant. Nevertheless, after selecting a certain biomarker, monitoring protocols should be established in oral and maxillofacial surgeons teams so as we have a correct understanding of biological values.

** Key words:**Serum biomarkers, oral cancer, diagnosis, prognosis.

## Introduction

Oral cancer is the sixth most common malignancy worldwide. Aproximately, 90% of cancer located in the oral cavity are oral squamous cell carcinoma (OSCC) (1). Most oral cancers are superficial and easily detected, but deeply located tumors may not be noted until they have grown large and reached an advanced stage. This malignant neoplasm occurs most commonly in the posteriorlateral border and ventral surfaces of the tongue. The second most common location is the floor of the mouth (2). Due their aggressiveness, oral cancer invades surrounding organs and causes regional or distant metastases (3). The overall survival rate for oral cancer is considerably lower than that of other cancers due to metastasis and recurrence (4).

Epidemiological studies showed variable incidences depending on the region. Incidence is particularly high in India, Brazil, Pakistan and France. Tobacco (particularly chewing) and alcohol have been large demonstrated as risk factors in the development of oral cancer (5). Additionally, these risk factors have been showed a synergist effect when they have been combined (6).

The determination of serum biomarkers is accepted as a valuable tool for diagnosis, finding therapeutic targets and prognosis in different kind of tumors (7). Literature has been showed overexpression in serum of some proteins (8), p53 antibody (9), and VEGF (8) as an indicator of oral cancer. Several biomarkers have been proposed, but they are sometimes variable with race, lifestyle, and carcinogen exposure. The global knowledge of all of them would lead to the improvement of diagnostic and prognosis methods of tumor recurrence and metastasis to assess changes in oral lesions (3).

-Serum biomarkers

Serum biomarkers are defined as substances changing quantitatively in the serum during tumor development. Classically, a marker is synthesized by the tumor and released into circulation or expressed at the cell surface in large quantity by malignant cells (10). These markers can been used in the prognosis of tumor recurrence or metastasis (11) because the development of the malignant tumor changing their concentrations (7). The tumor marker/substance can be classified as tumor specific and tumor associated. Tumor specific substance are considered as a direct result of oncogenesis, while tumor associated marker are various proteins, enzymes, hormones and immunoglobulins which occur in the blood and are mediated by the tumor itself or by the influence of the tumor on the involved tissues (12). Repeating test of serum biomarker allows following treatment and assessing response to treatment, monitoring tumor progression and metastasis (13). However, there are not yet unified parameters to determinate which biomarker would be useful for oral cancer.

The main focus of this systematic review is to analyze the utility of serum biomarkers in the diagnosis and prognosis of oral cancer.

## Material and Methods

-Search Strategy and Selection criteria

A systematic, computerized database search was conducted using the National Center for Biotechnology Information (NCBI) to search MEDLINE (Pubmed). The search was conducted using the following MeSHterms:”mouth neoplasms” AND marker AND (serum OR blood) [Mesh].

For the initial selection, article titles and/or abstracts were analyzed and the following inclusion criteria were observed: studies published in English in the last five years; studies of human beings; specific studies that analyzed a certain biomarker in serum; and study type: cases and controls studies, prospective and/or retrospective clinical studies. The exclusion criteria were: studies which do not mention the measurement method, studies that analyses markers in saliva.

**Figure 1:**
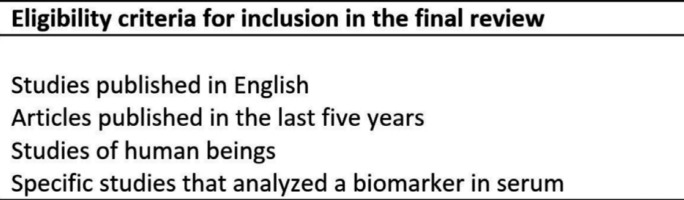
Eligibility criteria for inclusion in the final review.

Following initial selection, we read the previously selected articles fully, applying the selection criteria (Fig. 1) to determine final inclusion or exclusion from the study.

-Quality rating

A methodological quality rating was performed according to the PRISMA statement criteria in order to verify the strength of scientific evidence in clinicaldecision-making. The classification of the risk of bias potential for each study was based on the criteria adopted by Clementini *et al.* (14) described as follows: random selection of the sample; definition of inclusion/exclusion criteria; follow-up reports; validated measurements; statistical analysis. A study that included all the criteria mentioned above was classified as having a low risk of bias; astudy that did not include one of these criteria was classified as having a moderate risk of bias; when two or more criteria were missing, the study was assigned a high risk of bias ( Table 1).

**Table 1:**
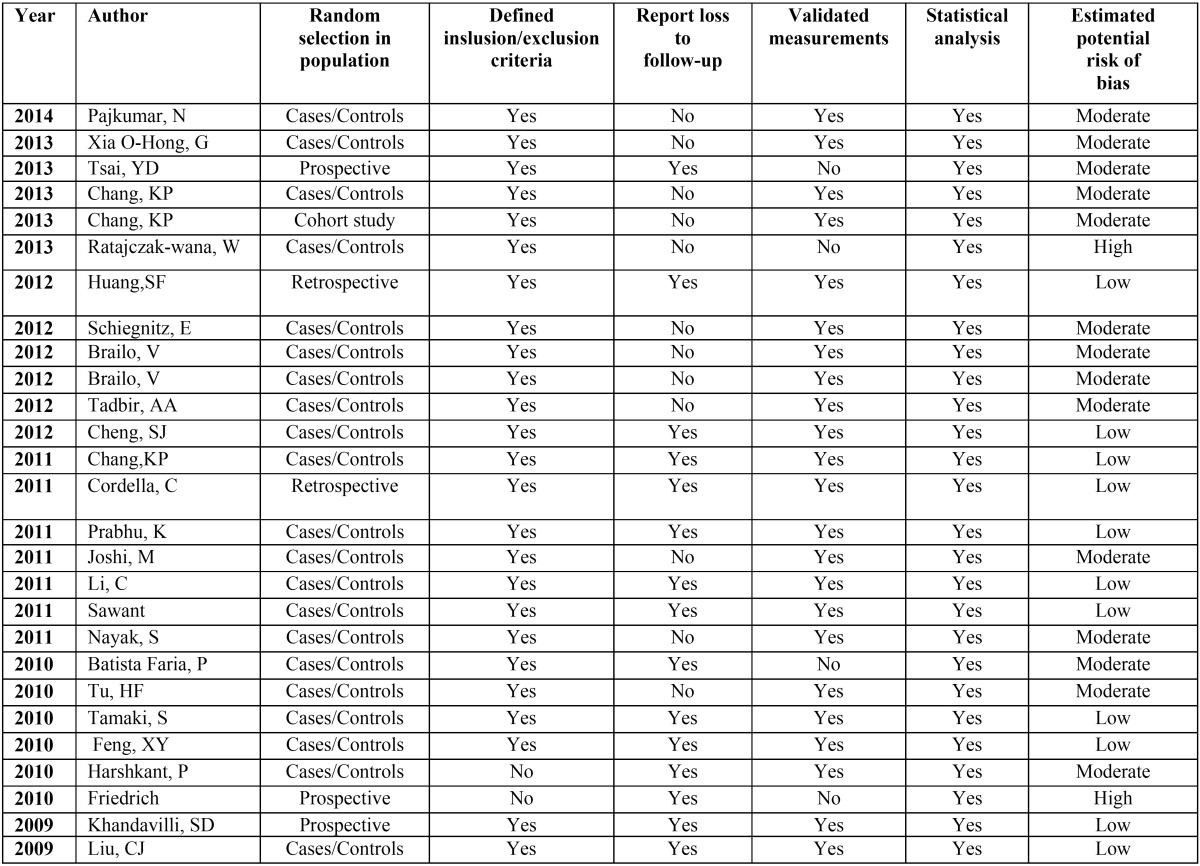
Quality assessment of the prospective and retrospective studies included.

## Results

**Figure 2:**
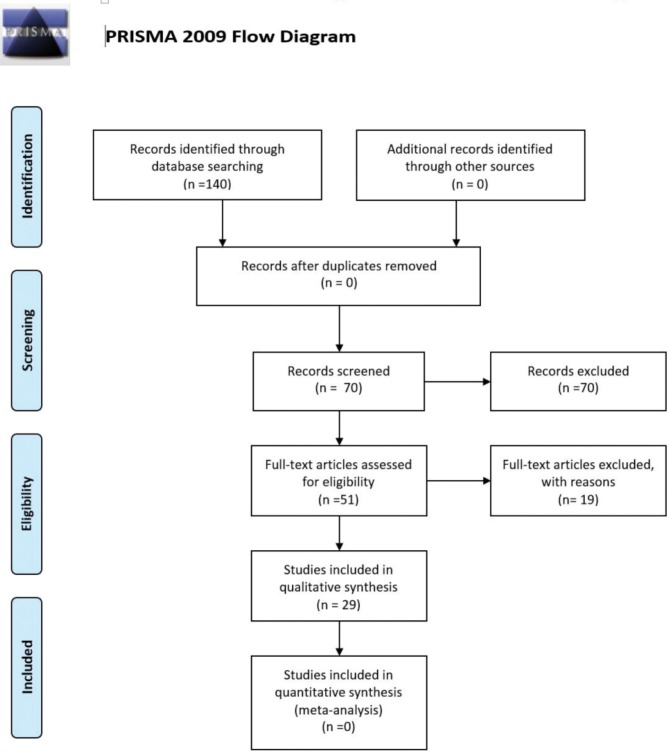
PRISMA Flow chart.

The electronic database search was performed on December, 2013 and yielded 130 results. Seventy articles were identified as relevant after reading the title and/or abstract. The full text of these 70 papers was evaluated according to the selection criteria in  Table 1. Of these 70 articles, six did not fulfill one or more selection criteria and were excluded. Twenty-seven articles were included in the final review. A flowchart of the selection and evaluation processes is shown in figure 2.

Of the articles included in the final review, twenty-two were cases/controls studies, one was a cohort study, two were prospective, and two were retrospective. The sample size of each cases/controls study ranged from 27 to 237 for patients and 14 to 112 for healthy controls ( Table 2, Table 2 continue).

**Table 2:**
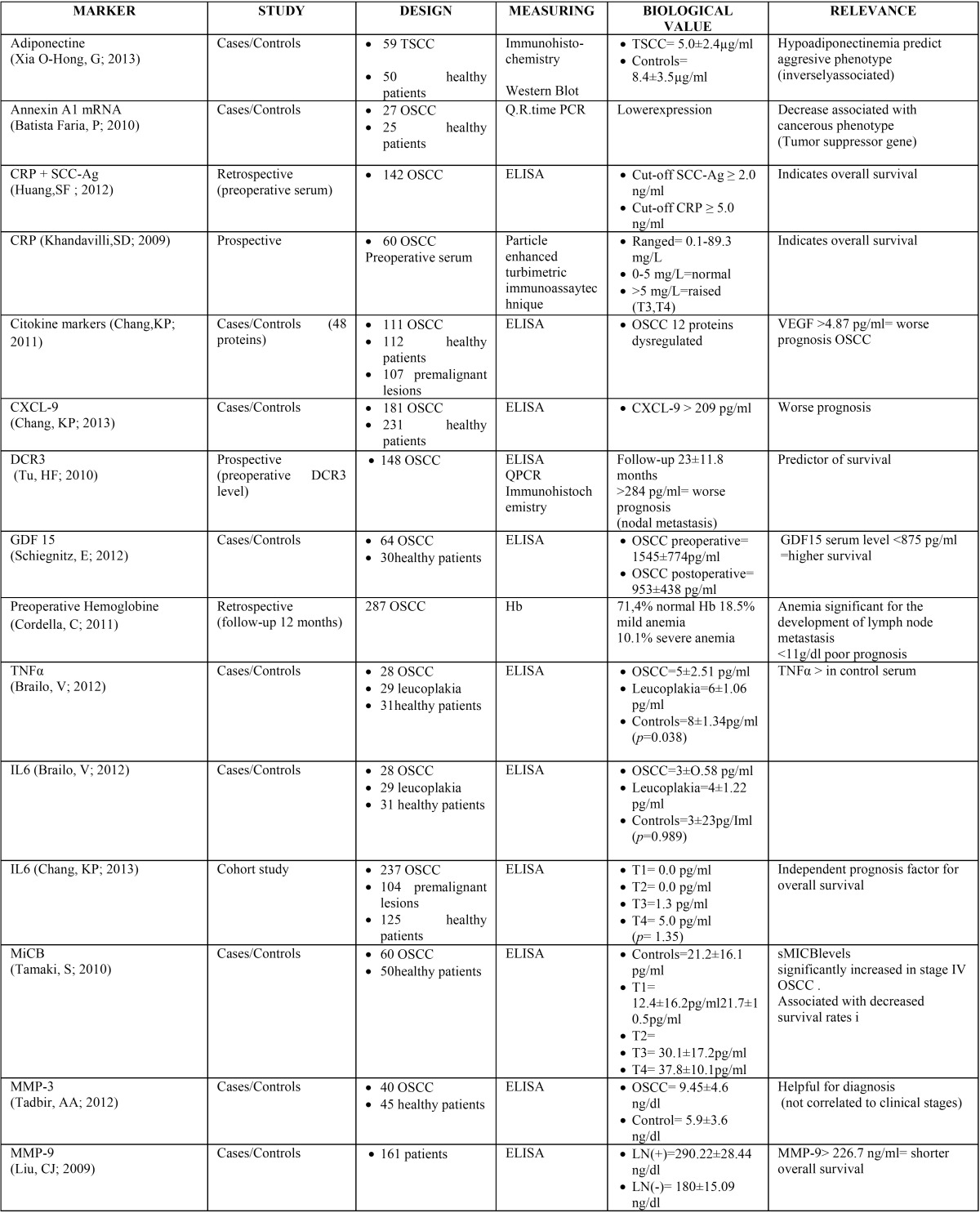
Summarize evidence on selected papers.

**Table 2continue:**
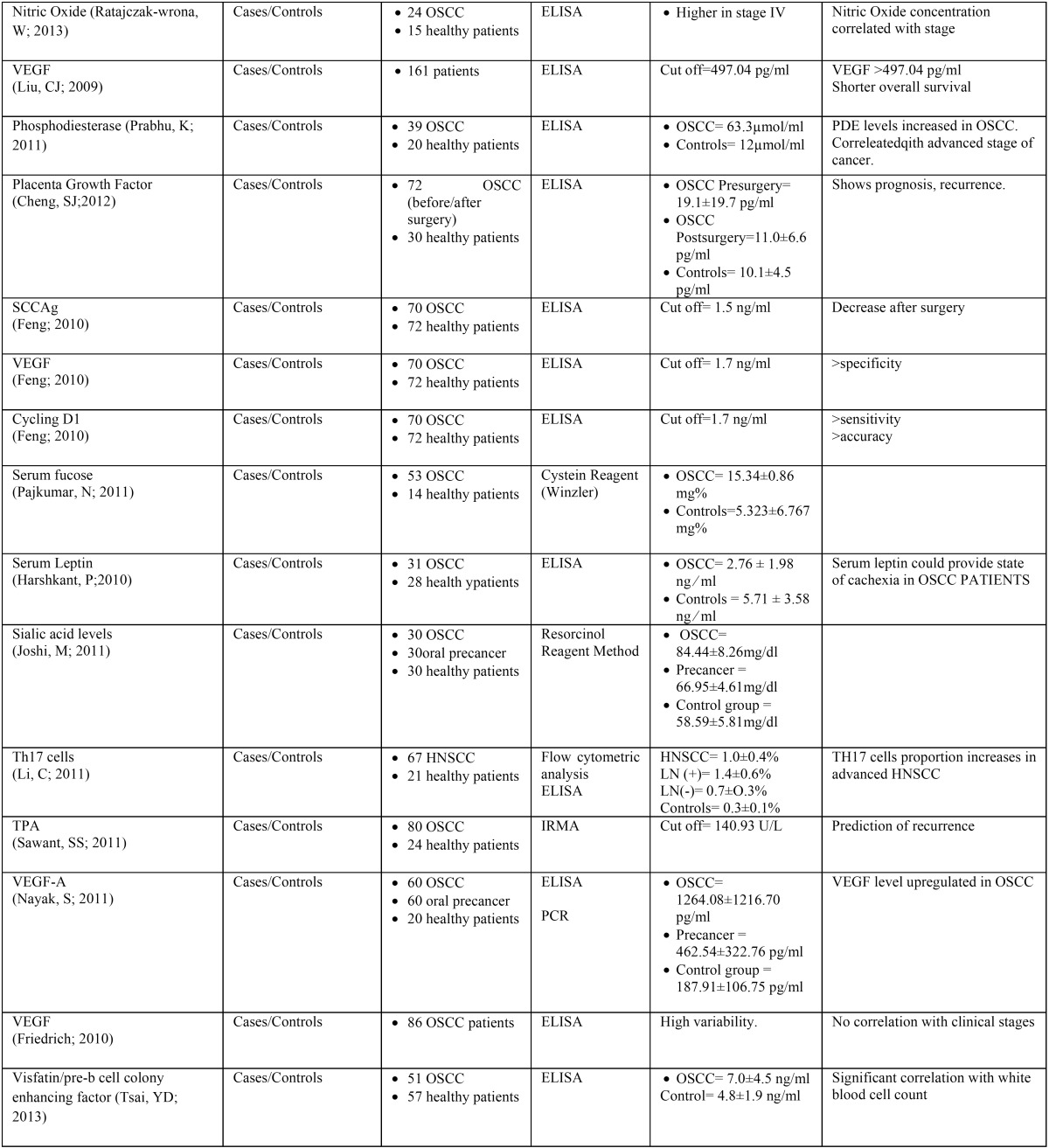
Summarize evidence on selected papers.

The most used measurement method was ELISA (enzyme-linked immunosorbent assay) in 17 studies, whereas other authors used Immunohistochemistry, Western Blott, flowcytometric analysis, IRMA (immunoradiometric assay), Resorcinol Reagent Method, Cystein Reagent method, particle enhanced turbimetric assay technique and PCR (Polymerase Chain Reaction) ( Table 2).

The twenty-six articles included in the final review reported twenty-two different biomarkers used in diagnosis and/or prognosis of oral cancer. Of those, four were specific tumor markers, direct result of oncogenesis, and twelve were associated tumor markers, substances mediated by the tumor ( Table 3).

**Table 3:**
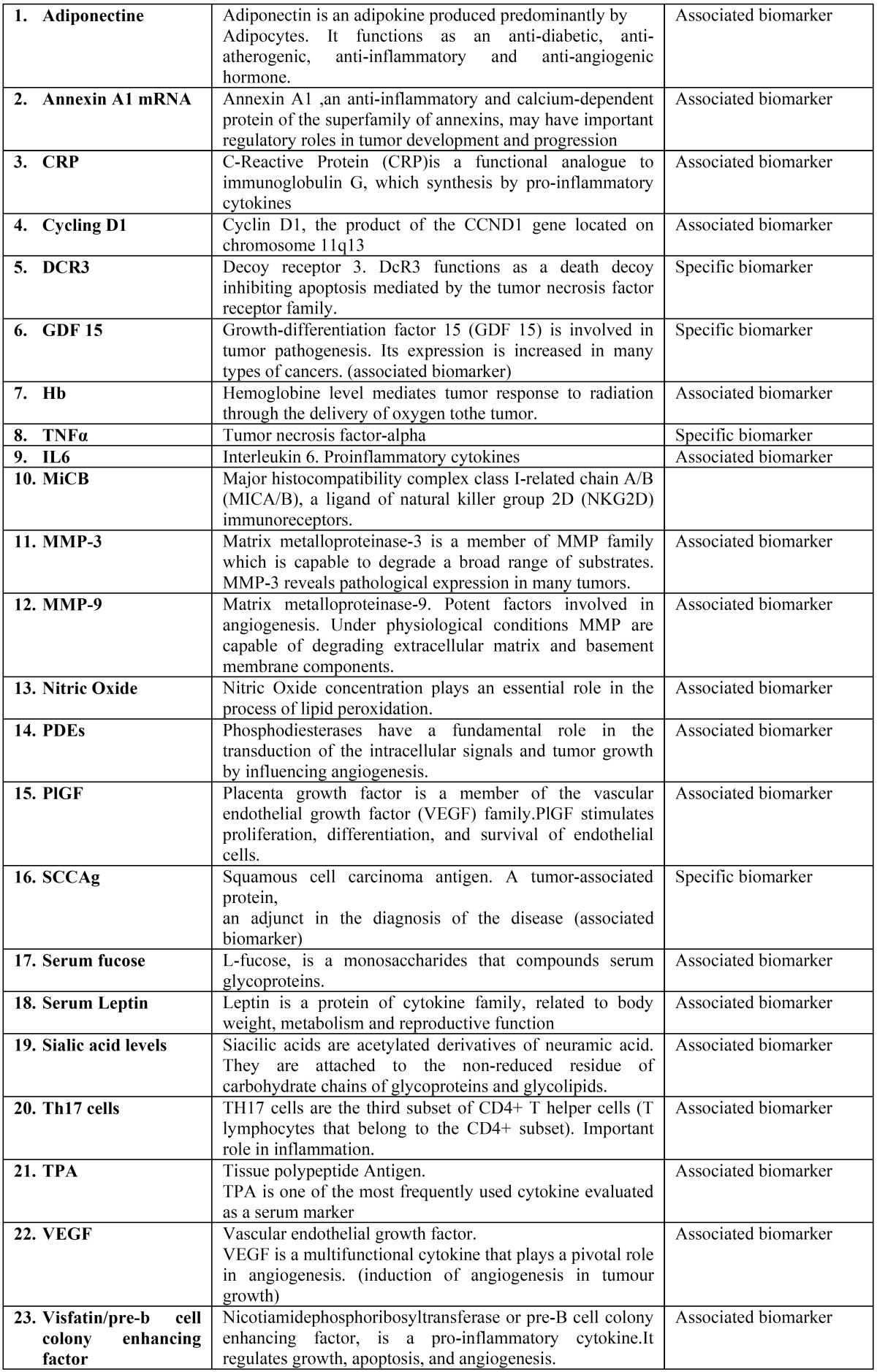
Biomarkers identified in studies.

Regarding the quality assessment, twelve studies achieved low risk of bias. Twelve studies were determined moderate risk and two studies were assigned to have high risk of bias ( Table 1).

## Discussion

Biomarkers have been wide accepted in other disciplines but there is no consensus for their use in oral malignancies. Despite recent advances in surgical, radiotherapy, and chemotherapy treatment protocols, the survival of patients with OSCC still lacks significant improvement. This unsatisfactory treatment may be explained by the fact that OSCCs frequently present with extensive local invasion and advanced stages (15,16). That makes necessary the development of new tools for the diagnosis and prognosis.

Tumor growth, invasion and metastasis are multiple step processes in which many genes and molecules are involved. The molecular biology of OSCC is complex and OSCC develops from the dysfunction of several interrelated pathways (17).

Our systematic review shows how several authors in the last years have looked for the best marker for diagnose oral cancer at earlier stages, establish the prognosis and increase the survival of patients with this disease.

-Adiponectine

Adiponectin is an adipokine produced predominantly by adipocytes that circulates abundantly in plasma and functions as an anti-diabetic, anti-atherogenic, anti-inflammatory and anti-angiogenic hormone (18).

In their study Guo *et al.* (19) showed that serum adiponectin level was reduced in tongue squamous cell carcinoma (TSCC), and inversely associated with histological grade and lymphnode metastasis of TSCC. They suggested that hypoadiponectinemia is correlated with histopathologic features of TSCC, and could be a new biomarker of aggressive phenotype in TSCC. But they still reckon the underlying mechanisms of adiponectin in potential cancer suppression are not fully elucidated.

-Annexin A1 mRNA

Annexin A1 an anti-inflammatory and calcium-dependent protein of the superfamily of annexins, may have important regulatory roles in tumor development and progression (20). The Annexin A1 gene expression was investigated by Faria *et al.* (21), in peripheral blood samples of patients with oral squamous cell carcinoma and control subjects and Annexin A1 mRNA was expressed in all of them. Comparative analysis of OSCC blood patients showed significantly lower Annexin A1 expression when compared to blood sample of control individuals. However, there were no significant differences between patients’ subgroups in relation to smoking, drinking, recurrence, TNM staging histopathological grading or therapies. This present study revealed the Annexin mRNA as new possible transcript biomarker for early detection of OSCC in the peripheral blood of patients.

-Cyclin D1

Xiao-yu Feng *et al.* (22) measured the level of some biomarkers (SCCA, Cyfra 21-1, epidermal growth factor receptor (EGFR) and Cyclin D1) in an attempt to determine the usefulness of their combined determination in the diagnosis of OSCC. They concluded that Cyclin D1, the product of the CCND1 gene located on chromosome 11q13, had the highest diagnostic specificity. Moreover the combined detection of EGFR and Cyclin D1 had the highest sensitivity, specificity and accuracy.

A previous study (23), demonstrates that Cyclin D1expression was significantly associated with the presence of occult lymph node metastases. These data suggest that the immunohistochemical analysis of Cyclin D1 expression in diagnostic biopsy samples may be an additional tool for selecting patients to be treated with elective neck dissection.

-C-Reactive Protein (CRP)

CRP is a functional analogue to immunoglobulin G, which synthesis by pro-inflammatory cytokines. An increase in the value of CRP has been demonstrated in patients with inflammatory disease and various cancers. In a recent study, Khandavilli *et al.* (24), investigated the relationship between preoperative serum CRP levels, tumor size, stage and survival for oral cancer patients. They found that two years survival rates in patients with preoperative elevation of serum CRP, more than 5mg/L, was significantly less favorable (44%) than that in patients without serum CRP elevation (90%). It demonstrated the link between raised CRP and malignant potential of oral SCC, concluding that it could be used as an independent prognostic indicator for patients with oral SCC treated by primary surgery.

-Decoy receptor 3 (DcR3) 

DcR3 functions as a death decoy inhibiting apoptosis mediated by the tumor necrosis factor receptor family. Frequently, gene amplification of DcR3 has been detected in various malignant tumors. Tu *et al.* (25) analyzed serum DcR3 level by an enzyme-linked immunosorbent assay (ELISA), quantitativepolymerase chain reaction (Q-PCR) and immunochemistry. They found that elevated serum DcR3 (>284pg/ml ) was associated with nodal metastasis and worse prognosis, concluding that serum DcR3 level is an independent prognostic factor of OSCC and also a predictor for neck nodal metastasis.

-Growth-differentiation factor (GDF 15)

Growth-differentiation factor 15 (GDF 15) is a member of the transforming growth factor-b (TGF-b) superfamily, involved in tumor pathogenesis and its expression is increased in many types of cancers (26). Schiegnitz *et al.* (27) reported for the first time, *in vivo*, enhanced serum GDF 15 levels in patients with OSCC and provided evidence demonstrating a significant relationship between serum GDF 15 levels and prognosis of the patients. However, they concluded the role of GDF 15 in cancer pathophysiology is not clear yet. The diagnostic utility of GDF 15 could be improved by combining GDF 15 with other serum markers.

-Hemoglobine (Hb)

Low Hb levels are indeed associated with poor tumor oxygenation and increasing Hb concentrations are correlated with higher pO2 levels and lower hypoxic tissue fractions. In a retrospective study, Cordella *et al.* (28) settled the hyphotesis that if a low Hb concentration is a predictor of decreased local control, Hb corrections may significantly improve tumor oxygenation and prognosis. They found that anemia was significant for the development of lymph node metastasis as well as for the development of local recurrence. Preoperative transfusion or erythropoietin administration before surgery has very important economic as well as physiologic consequences so this idea should be considered with caution.

Further investigations are needed in a prospective setting, with greater evidence, to rule out dependency with other more important factors.

-Cytokines

Proinflammatory cytokines interleukin 1 beta (IL-1β), interleukin 6 (IL-6) and tumor necrosis factor alpha (TNF-α) regulates inflammatory response and play significant role in the development of cancer (29).

In their study, Brailo *et al.* (30) showed that patients with oral cancer have higher salivary IL-1β and IL-6 concentrations compared to patients with leukoplakia and healthy control but no significant differences in serum IL-6 were observed between the groups. However, serum TNF-α concentration was significantly higher in control subjects compared to oral cancer patients.

Chang *et al.* (16) conducted a study to demonstrate the possible biologic relevance of potential cytokine markers in OSCC. They analyzed the associations between the clinicopathologic manifestations of OSCC and the blood levels of the 12 individual cytokines. As Brailo *et al.* (30) did before, they find strong associations between some increased cytokine levels and clinical factors but the study did not reveal any associations between others cytokines with elevated levels in OSCC patients and clinicopathologic manifestations.

These investigations fails to identify certain cytokines or cytokine panels that could be used to effectively detect OSCC patients, Results from this studies and heterogeneous literature data indicate that altered cytokine production and responsiveness in oral cancer takes place primarily in the oral cavity and does not reflect on serum cytokine concentrations

-Major complex class I-related chain A/B (MIC-B)

Expression of MIC-A/B, ligands of natural killer group 2D, has been proposed to play an important role in tumor immunosurvei-llance. Soluble forms of MICA/B are increased in sera of cancer patients and are postulated to impair antitumor immune response by down regulating expression of NKG2D immunoreceptors. In advanced stages of some tumors have been reported increases in soluble MIC-A (31). Watson *et al.* (31) found that OSCC patients with high soluble MIC-B levels had significantly lower survival rates. Furthermore, patients with both high soluble MIC-A and soluble MIC-B levels also had markedly decreased survival rates.

Tamaki *et al.* (32) reported that serum MICB levels did not differ significantly from those in normal control individuals. However, they indicated that serum MICB levels were significantly increased in stage IV OSCC and it was significantly associated with decreased survival rates in patients. These findings suggest the utility of sMICB levels as a marker for tumor progression.

-Matrix metalloproteinase enzymes (MMPs)

MMPs are proteolytic enzymes and in cancer they regulate various cell behaviors by degradation of proteins. These include cancer cell growth, differentiation, apoptosis, migration, invasion and regulation of tumor angiogenesis and immune surveillance.

Liu *et al.* (33) analyzed the association between pretreatment serum levels of MMP-9 and clinic-pathological parameters and outcome for patients with OSCC. In this investigation patients with MMP-9 serum levels higher than median (226.7 ng/mL) had significantly shorter overall survival than those with levels lower than median. It suggested pretreatment serum levels of MMP-9 as a powerful prognostic marker in patients with oral squamous cell carcinoma.

Tadbir *et al.* (34), analysed serum MMP-3 level in OSCC patients. Their results showed that serum MMP-3 level in OSCC patients was significantly higher than healthy controls but they couldn`t correlate serum MMP-3 concentration with the clinicopathological. Unlike the previously mentioned study, the results suggest that the measurement of serum MMP-3 concentration might be helpful to diagnose OSCC but not to predict prognosis.

-Squamous cell carcinoma antigen (SCC-Ag)

SCC-Ag, a tumor-associated protein, was first isolated as “TA-4” from SCC tissue of the uterine cervix in 1977 (35). Since then, several studies have shown that serum SCCA was elevated in OSCC patients and could be used as an adjunct in the diagnosis of the disease. Recently some studies have found that serum SCC-Ag concentrations were significantly increased in OSCC patients, and that the SCC-Ag level decreased significantly after tumor resection (22).

SCC-Ag serum level was also correlated with tumor. Moreover other investigations mentioned it may be a useful tool for monitoring the course of the disease and its recurrence (22).

These studies shows evidence enough to remark the utility of SCC-Ag, a specific antigen, in the diagnosis and prognosis of oral cancer if serum levels are well controlled during preoperative and the follow-up.

-Sialic acid level

Siacilic acids are acetylated derivatives of neuramic acid. They are attached to the non-reduced residue of carbohydrate chains of glycoproteins and glycolipids. Altered glycosylation of glycoconjugates is among the important molecular changes that accompany malignant transformation (35) .

Joshi (12) found the mean serum total sialic acid levels in control group (58.59±5.81mg/dl), oral precancer (66.95±4.61mg/dl) and oral cancer group (84.44±8.26mg/dl) were statistically significant (p<0,005). These differences were also found by Rapjura *et al.* (36) (control group=30.25±2.49mg/dl; cancer group=63.70±19.40 mg/dl). Sialic acid level is directly proportional to tumor burden (35,36). Joshi (12) found the mean serum total sialic acid levels in stage I was 71,24 mg/dl whereas it was 73,36 ±4,65 mg/dl, 84,61±6,40 mg/dl, and 89,34± 4,68 mg/dl in stage II, stage III and stage IV respectively.

-T(h)17

TH17 cells are the third subset of CD4+ T helper cells (T lymphocytes that belong to the CD4+ subset), which are characterized by their production of interleukin (IL).

17A and IL-17F have been verified to play an important role in inflammation, autoimmune diseases, and human organ transplantation rejection. Li *et al.* (37), reported an increase of serum IL-17 levels in patients with head and neck squamous cell carcinomas (HNSCC) compared with healthy control subjects (123.35-45.13 pg/mL vs. 20.78-3.95 pg/mL; *p*<0.05). The results indicated that IL-17 expression can be detected in the very early stage of squamous cell carcinoma and increases gradually with the development of the tumor. There was significant difference between TH17 cell proportions in peripheral blood in patients with or without lymph node metastasis. This study suggested that TH17 cells may be involved in tumor growth and metastasis of HNSCC.

-Tissue polypeptide Antigen (TPA)

TPA is one of the most frequently used cytokine evaluated as a serum marker for its clinical applications. In their study Sawant *et al.* (38), using immunoradimetric assay, found that elevated levels of TPA was correlated significantly with stage (*p* = 0.02), development of recurrence (*p* < 0.006), and impacted survival (*p* < 0.033). This result indicates that TPA can be a useful tumor marker for the prediction of recurrence and poor prognosis in human oral cancer.

-Vascular endothelial growth factor (VEGF) 

VEGF is multifunctional cytokine that plays a pivotal role in angiogenesis. It has been considered as the most potent one for the induction of angiogenesis in tumor growth. Shang *et al.* (39), determinate that serum VEGF concentration was increased in patients with OSCC (Control group=148.80±64.17pg/ml, Cancer=567.97±338.17 pg/ml. *P*<0.001) Increased values of VEGF has been found with progression of disease and decreased values after surgery. Higher level of serum VEGF was closely associated with lymph node metastasis 33 and clinical stage in OSCC patients (33,39). Finally, elevated serum VEGF levels have been correlated with poor disease-free survival and poor progression-free survival in cancer patients (33).

We have found quite homogeneous criteria and protocol to investigate the role of serum biomarkers but there is still no unified criteria for using a certain marker or another.

Our results highlight that a wide variety of biomarkers have been studies and a great part of theme have demonstrated their effectiveness in the diagnosis and/or prognosis of oral cancer. Most of the investigations are cases and controls studies where the measurement chosen system is ELISA. Surprisingly, the quality of the articles included was acceptable and were classified as “low risk of bias”.

The main limitation of the studies in our systematic review is that there is no a real follow-up of the patients and they do not repeat all the measurements in serum. We think this is crucial to correlate biological values with the progression and prognosis of the disease so future investigations should contemplate this item to provide more evidence of the utility of serum biomarkers.

Biomarker use for diagnosis and prognosis is supported by clinical and scientific evidence is relevant. Nevertheless, after selecting a certain biomarker, monitoring protocols should be established in oral and maxillofacial surgeons teams so as we have a correct understanding of biological values.
